# Preparing for COVID-19 exit strategies

**DOI:** 10.1016/j.amsu.2020.12.012

**Published:** 2020-12-13

**Authors:** Michelle Griffin, Catrin Sohrabi, Zaid Alsafi, Maria Nicola, Ahmed Kerwan, Ginimol Mathew, Riaz Agha

**Affiliations:** a Stanford University, Palo Alto, United States; b Barts and The London School of Medicine and Dentistry, Queen Mary University of London, United Kingdom; c Imperial College Healthcare NHS Trust, London, United Kingdom; d The Lister Hospital, East and North Hertfordshire NHS Trust, United Kingdom; e UCL Medical School, University College London, United Kingdom; f Barts Health NHS Trust, London, United Kingdom

**Keywords:** Coronavirus, COVID-19, Exit strategy, Reopening, Flatten the curve, Social distancing

## Abstract

The COVID-19 pandemic has affected 20 million people worldwide with over 732,000 deaths and trillions of dollars of lost economic productivity. It has put many countries into lockdown to contain the virus and save lives. As COVID-19 cases in some countries start to plateau and societies work hard to ‘flatten the curve’, leaders are being asked to formulate plans for safe and staged ‘exit strategies’ to reopen society. Each country will decide on their own exit strategy but many plans are considering similar vital healthcare principles including the maintenance of social distancing to prevent ongoing community transmission, testing capacity, protection of the healthcare systems and the health of their care workers. This review aims to provide an overview of essential factors that plans for exit strategy should consider and their effect on the societies' social and healthcare life.

## Highlights

• Exit strategies following COVID-19 are being formulated with guidelines to ensure society returns to ‘normality’ safely.
• Exit strategies are taken a phased approach to end lockdown following COVID-19.
• Exit strategies should consider ongoing community transmission, testing capacity and protection of the healthcare systems.

## 1 Introduction

The novel Coronavirus SARS-2 (COVID-19) was first announced in China on the December 31, 2019, with the World Health Organization (WHO) declaring a public health emergency on January 30, 2020 [1]. On the 11th March it was classed as a pandemic, with the virus in over 60 countries with profound effects on societies' social and economic life [2]. To contain the virus many countries were locked down to reduce the spread of the virus, decrease the load on health services and save lives [2]. While lockdown may contain the virus, governments cannot continue lockdowns indefinitely and require ‘exit strategies’ to unlock cites to restore a degree of ‘normality’ [3]. With no proven treatment for COVID-19, governments must implement advice on physical distancing, quarantine and travelling [3]. However, how each government devises and executes exit strategies is varied [3]. Many countries are considering several overlapping health principles including, social distancing rules, testing capacity and protection of health care systems [3]. With limited evidence guiding governments on the optimal way to contain the virus, many countries have taken the approach to slow the spread by aiming to ‘flatten the curve’. [3]. This literature review aims to provide an overview of current COVID-19 exits strategies implemented to reopen society safely including timing, testing capacity and social distancing. A discussion on the key health care principles that should be considered for re-opening is also provided. Furthermore, a brief overview on the current status of vaccine development for overcoming the COVID-19 pandemic is provided.

## 2 Considering the timing of the exit strategy

The timing to open society is of utmost importance when considering an optimal exit strategy. Many societies have been seen considering opening when they have reached the ‘post peak period’ [3,4]. Although there is no definition for the ‘post peak period’ to date, experts have shown to agree that a plateau of cases for two weeks may show that health care systems can cope with the number of COVID-19 cases [3,4]. However, the timing of reopening cannot be assumed to be the same among all countries, with governments needing to take into account individual healthcare, economic and social considerations. Following the decision to open society, leaders must judge on the most optimal staged approach [5]. Many societies have taken the approach to open lockdown in ‘phased approach’ [5]. The American Enterprise Institute, published a report on March 29th, 2020 entitled ‘*National Coronavirus Response: a Road Map to Reopening*’, to provide guidance on formulating a phased approach [5,6]. The report showed four phases whereby each phase must be completed before the next is reached. Phase I uses community-level physical distancing measure to slow the spread, asking communities to stay at home to ‘slow the spread’ [5]. During this phase diagnostic testing and public health facilities should be expanded to allow health care systems to treat the virus [5]. A shift to phase II is allowed when 4 criteria have been met including, (1) the number of new cases has declined for at least 14 days, (2) rapid diagnostic testing is sufficient to test all people with COVID-19 symptoms, (3) healthcare systems are able to safely care for all patients, and (4) public health capacity allows for effective contact tracing [5]. During phase II, some businesses may begin reopening with COVID-19 measures in place. Phase III is considered the time when effective therapeutic vaccines are available [5]. Lastly, Phase IV, is when society reflects on their preparedness for tackling the next health threat [5]. Although many countries are implementing similar staged approaches, plans for opening of schools and businesses, travel restrictions, public facilities and transport will be specific for each government.

## 3 Testing capacity during the exit strategy

One of the most important considerations during the creation of an exit strategy is devising a plan for COVID-19 testing. Amidst the calls by WHO who emphasizing the importance of testing and calls for countries to increase their testing capacity, a group at Imperial College London have investigated the effect Healthcare worker (HCW) and at-risk group testing may have. Using mathematical modelling, they estimate that weekly selective testing of these groups alone regardless of symptomatology can decrease the transmission of the virus by 25–33% more than self-isolation alone. Furthermore, the group has claimed that testing the general population will not significantly contribute to limiting transmission more than contact-tracing and quarantine alone. They conclude that testing is important for pandemic surveillance but prevention of spread is more likely to affect high-risk individuals, patients and HCWs [7].

On a pragmatic scale, although the cost-benefit analysis of each individual test may not be favorable, it is now evident that as a result of mass testing, countries like South Korea have managed to maintain a better control of the virus. It is therefore prudent for efforts to be made in order to deliver testing “on a reliable, affordable and inclusive basis” ensuring testing is available for individuals of all socio-economic backgrounds. Vietnam's efforts to contain the virus despite limited resources has been praised internationally. Specifically, excellent communication, enforced quarantine and affordable test kits remained the countries main focus throughout the outbreak. Collaboration between government, the private sector, hospitals and university researchers as well as utilising public funding, the state was able to produce cheap testing kits that produced results in less than 2 hours. Vietnam's early and decisive actions to prioritise COVID-19 testing, instigated a sense of collaborative effort, maximising the speed of innovation-to-production and hence reaching a large testing capacity [8].

In the UK, issues arose regarding implementation of testing and reaching daily testing targets despite the country's high testing capacity. The decision to centralise all testing took too long to implement, an issue not faced by Germany or South Korea who opted for localised testing centres. With all focus being set on delaying and mitigating the disease, poor coordination and strict testing criteria, the UK failed to utilise all available laboratories for COVID-19 testing [9]. Testing in the UK did increase dramatically, from 2000 tests per day in March to over 100,000 by the beginning of May, after decentralising the testing approach and involving NHS laboratories, private testing providers and academic institutes, an approach similar to Vietnam's mentioned above. The UK government has pledged to perform 200,000 tests per day by the end of May, [10,11].

## 4 Maintenance of social distancing during the exit strategy

In response to the Covid-19 pandemic, countries around the world have instituted lockdown to uphold social distancing. Indeed, it is primarily contact with other humans which allows SARS-CoV-2 to spread. However, lockdown has also caused significant economic and social disruption [12] compelling governments to trial exit strategies whilst the virus is still spreading – albeit at a lower rate. In order to avoid a second wave during the exiting of lockdown, maintenance of social distancing is crucial [13].

As governments around the world are slowly encouraging a return to normality, health agencies strongly encourage employers to differentiate between essential and non-essential workplace presence [14]. Employers should put in a concerted effort to facilitate working from home wherever possible. Examples of this would include providing the necessary equipment needed to work safely and effectively whilst remote. Indeed, large companies such as Google and Facebook have already announced their support of employees to work from home until the end of the year [15]. In the interest of employers required to be present in the workplace, public transport officials should be advised to facilitate social distancing (e.g., keeping commuters 2 m apart on carriages). Studies have demonstrated the ability of the virus to persist on a range of surfaces, thus the frequency of carriage decontamination should be increased to multiple times per day [16]. Once in the workplace, social distancing where possible should be upheld and regular hand washing encouraged.

The re-opening of non-essential retailers poses a dynamic risk which must be constantly monitored. Shops should determine the volume of customers their floorspace can accommodate whilst allowing 2 m of distancing. Furthermore, areas at particular risk of congestion (e.g., doorways, around the cashier etc) should be subjected to further limitations. The UK government has advised businesses to suspend or reduce customer services which contravene social distancing guidelines [17]. For example, a pair of workers should be called to lift a heavy object rather than a single worker lifting with a customer [17].

## 5 Surveillance of COVID-19 in society during the exit strategy

The implementation of restrictive measures has been necessary to help delay the spread of COVID-19 and to alleviate mounting pressures on health care systems worldwide. Following the relaxation of confinement measures however, appropriate monitoring capacity is required. These include large-scale testing services to facilitate the detection of viral components to monitor the extent of disease prevalence and spread. Testing can be combined with contact tracing, which enables the contacts of infected individuals to be traced and monitored as well as to notify them of their exposure. This additionally serves to support the quarantine of contacts, ensuring safe, sustainable, and effective quarantine. This time-tested public health strategy is standard with infectious diseases, having been implemented during the 2003 SARS outbreak and 2014 Ebola outbreak. More recently, the UK NHS test and trace service has been deployed [18]. However, care should be taken when establishing such services, as recently demonstrated following the exposure of security weaknesses in Qatar's mandatory coronavirus contact-tracing app [19]. Surveillance can additionally be mediated through antibody detection capacities, which will provide complementary data on the proportion of the general population having attained a degree of acquired immunity.

The initial, gradual restoration of health services is likely to focus on those of high urgency, including those for cancer and mental health [20]. Overall, services likely to be prioritised following the lifting of restrictions include cancer care, acute cardiac surgery and hospital services for percutaneous coronary intervention, interventional neuroradiology for mechanical thrombectomy, secondary care services including urgent arrhythmia and severe heart failure or valve disease, and prioritization for stroke services.

## 6 Ongoing clinical trials for the COVID-19 vaccine

As of August 11, 2020, there have been over 20 million confirmed cases of COVID-19 worldwide, with over 700,000 lives lost [21]. A vaccine against COVID-19 is often considered to be the only viable long-term solution for this pandemic [22]. However, vaccine development is a lengthy process, often taking over 10 years [23]. With more human lives at stake, along with grave socioeconomic implications, a vaccine against COVID-19 needs to be developed considerably quicker [12,24]. As of August 12, 2020, the WHO has listed over 100 potential COVID-19 vaccine candidates, at different stages of development; currently, the goal is to develop a COVID-19 vaccine in 12–18 months [25,26].

Traditionally, vaccine development is a complex process, spanning several years and involving multiple stages. First is the exploratory stage, which spans 2–5 years and involves laboratory-based research aimed at identifying natural or synthetic antigens of a pathogen that could be used to prime the immune system for future encounters with that pathogen. The next stage is the preclinical stage which spans 2 years and involves the assessment of safety and immunogenicity of vaccine candidates in cell culture and animal models. Thirdly is the clinical development stage, which spans 2 years, where vaccine candidates are tested in humans for the first time. In the Phase 1 clinical trial, the safety and tolerability of vaccine candidates are evaluated in a group of 20–80 people, usually healthy individuals. Phase I studies can be open label unlike phase II and III studies which are usually double-blind, randomized and controlled. Phase II, spans 2–3 years and involves the assessment of safety, immunogenicity and dose ranging of vaccine candidates, in several hundreds of people, with characteristics resemblant of the target population. Phase III, spans 5–10 years and involves the assessment of safety and efficacy of vaccine candidates, in several thousands of people, representative of the target population. Following the clinical trial there is a regulatory review and approval stage which spans around 2 years and involves the submission of all available data (pre-clinical and clinical) to regulators, who will scrutinize the safety and efficacy data and carry out a risk/benefit assessment, to decide whether to approve or refuse approval for a vaccine candidate. Following this is the manufacturing stage where the production of approved vaccine candidates is scaled up to commercial levels. Following regulatory review and approval, regulators oversee the production and manufacturing process of approved vaccine candidates to ensure manufacturers are able to produce vaccines safely and reliably [2,23,26].

In order to achieve the target of a COVID-19 vaccine in 12–18 months, the traditional vaccine development process, spanning over 10 years, has been/can be sped up in several ways [27]. Firstly, the genetic sequence of COVID-19 was publicly shared, early on in this pandemic. On January 12, 2020, China publicly shared the genetic sequence of the causative organism, under two months since the supposed beginning of this pandemic in early December 2019. Hence, researchers across the globe were able to rapidly commence the exploratory stage of vaccine development [[28], [29], [30]]. Secondly, lessons learned from previous coronavirus outbreaks (i.e. severe acute respiratory syndrome (SARS) outbreak in 2002/2003 and Middle East respiratory syndrome (MERS) outbreak in 2012) – vaccine development against both of these coronaviruses had begun but was left unfinished upon successful containment of both of these outbreaks. However, researchers can draw on these previous experiences of developing vaccines against coronaviruses to guide the development of a COVID-19 vaccine [30]. Several novel vaccine technologies have been implemented including vaccine platform technologies (e.g., nucleic acid-based vaccines and viral vector-based approaches) to speed up development and manufacture [31]. Furthermore, having different stages of vaccine development process concurrently has allowed vaccine development to be carried out in a sequential manner. In response to this pandemic, vaccine development is allowed to progress to human testing before animal testing is completed. Remaining stages of animal testing can continue alongside the trialing of vaccine candidates in humans or certain stages of animal testing can be skipped [30]. In addition, different phases of the clinical development stage can be shortened and partially overlapped [32]. Rolling review has been implemented, which is where data can be submitted to the regulators as they become available, allowing assessment of data on a rolling basis allowing the regulatory review and approval to be fast-tracked [33]. Furthermore, investing in the manufacturing of vaccine candidates throughout the vaccine development process rather than after approval has allowed the building of facilities required to manufacture the most promising vaccine candidates as well as scaling up the production of the most promising vaccine candidates before approval will ensure the availability of sufficient doses as soon as a vaccine candidate becomes approved. However, this approach comes with the financial risk of vaccine candidates failing to secure regulatory approval [26,34]. Human challenge studies have also been used, where deliberate infections of volunteers occurs instead of waiting for them to become infected in the community in order to swiftly obtain information regarding the efficacy of a vaccine candidate. However, human challenge studies raise ethical issues as healthy volunteers are put at risk of serious illness or death in the absence of a proven effective treatment [35]. WHO has published ethical guidelines for performing human challenge studies during COVID-19 [36].

As of August 11, 2020, 28 vaccine candidates have entered the clinical development stage of the vaccine development process [[25], [37], [38], [39]]. More recently, on November 18th^,^ 2020, Pfizer and BioNTech announced that they have developed a COVID-19 vaccine that is 95% effective in patients without previous COVID-19 infection [40]. The Medicines and Healthcare products Regulatory Agency (MHRA) in the UK have already allowed the vaccine to be used and have plans to begin implementing vaccinations [41]. Once a vaccine becomes fully available, sufficient uptake of the vaccine by the public is fundamental to help ending the pandemic. A poll conducted by the Royal Society for Public Health showed that one in five people, in the UK, would not get vaccinated against COVID-19 or were not sure if they would; a poll conducted by Morning Consult revealed that only 30% of people in the US would want to get vaccinated, soon after the vaccine becomes available [42,43]. Estimates suggest that between 55% and 82% of the population need to be immune against COVID-19, determined by varying environmental, biological and socio-behavioral factors, in order to achieve herd immunity and hence disease control; assuming that a certain proportion of individuals will have contraindications to vaccinations, a vaccine rejection rate >10% could jeopardize the success of a vaccine against COVID-19 [44].

According to the Coalition for Epidemic Preparedness Innovation (CEPI), COVID-19 vaccine development will cost $2 billion; CEPI is an international non-governmental organisation set up in 2017, to support the development of vaccines against emerging infectious diseases, funded by the Bill & Melinda Gates Foundation, the Wellcome Trust, the European Commission and eight countries (i.e. Australia, Belgium, Canada, Ethiopia, Germany, Japan, Norway and UK) [45,46]. Several countries contributed, in response to CEPI's call for $2 billion to support COVID-19 vaccine development and CEPI has raised $1.4 billion so far [47]. Plans need to be put in place to ensure global access to COVID-19 vaccines and to prevent rich countries from monopolizing the global supply of vaccines as observed during the influenza A/H1N1 pandemic in 2009 [48].

## 7 Restoration of healthcare services during the exit strategy

Before resuming elective services, a number of key facility and logistical considerations should be factored in, including timing, testing, personal protective equipment and local co-ordination [49]. Timing is very important, a sustained reduction in the rate of COVID-19 presentation should be observed, to enable the availability of sufficient staff. Testing is of utmost importance, a clear diagnostic testing availability should be in place within each hospital to ensure adequate testing for staff and patients when needed. Considerations regarding personal protective equipment are needed, for example, adequate PPE and surgical supplied should be available. There must be local co-ordination within the healthcare set up to ensure that the patients’ care pathway is appropriately managed, safe and efficient, and not compromised in any way.

Guidelines reporting the levels of surgical priority have previously been published [[50], [51]]. Considering there has been an overwhelming number of non-urgent elective operations postponed since the start of the COVID-19 pandemic, global healthcare systems will likely face a long if not indefinite crisis to alleviate this building backlog [52]. It is likely that the priority of resumption of surgical work will follow on from prior guidelines having detailed the level of urgency of a given surgical procedure.

## 8 Behavioral and cultural shift that COVID-19 will introduce in the healthcare systems duirng the exit strategy

The COVID-19 pandemic has forced governments to question the integrity of their healthcare systems and has expedited behavioral and cultural shifts within healthcare that would ordinarily take decades to achieve.

The value of digital health was highlighted during the pandemic, with the use of telemedicine skyrocketing [52]. In the UK, hospital and general practice appointments have been supplemented or substituted with video and telephone consultations and the number of patients requesting prescriptions online has increased by 97% [52]. The use of these solutions along with innovations in drones and robotics are likely to continue, as patients realize, they have more digital options that can accommodate their needs. More controversially however, various governments around the world -ranging from Singapore to Israel-have tracked and traced the spread of the infection using citizen's mobile data. Concerns have been raised as this may set a dangerous precedent regarding data protection and personal privacy [53].

Governments, the WHO and various charities have also invested heavily in educating the public on hygiene measures such as the importance of regular handwashing and social distancing. One would hope that these standards will continue to be upheld as they are vital in preventing the spread of COVID-19 and future pandemics.

In addition, prior to the pandemic, it was estimated that approximately 50% of the world's physicians experienced burnout [54]. The pandemic will inevitably lead to increased rates of burnout among healthcare professionals who have faced shortages in PPE, worked prolonged hours and witnessed the deaths of countless patients, colleagues and loved ones [54,55]. Now and when the dust settles, it is vital that we find and develop sustainable healthcare models that not only look after patients, but also the health and wellbeing of the healthcare professionals that keep the engine running.

## 9 Conclusion

Each governmentwill decide, on an individual basis, how to open society following the enforced lockdown from COVID-19. Many reopening strategies have considered a staged approach to slow the virus. Furthermore, with considerations of key healthcare principles including the maintenance of social distancing, testing capacity, ongoing vaccine trials and support for healthcare systems, societies have been able to open safely from lockdown. With many approaches being taken by different governments, we will learn with time what measures are optimal to contain the virus in a safe and effective manner. Considering the immense challenge facing governments to re-open safely it will be important to share successful approaches and potential roadblocks to ensure we face the burden of COVID-19 together.

## Funding

None.

## Provenance and peer review

Not commissioned, externally peer-reviewed.

## Author contribution

MG, CS, ZA, MN, AK, GM, RA all contribute to study design and wrote the manuscript.

## Declaration of competing interest

None.
